# An Assessment of Health Risks and Mortality from Exposure to Secondhand Smoke in Chinese Restaurants and Bars

**DOI:** 10.1371/journal.pone.0084811

**Published:** 2014-01-08

**Authors:** Ruiling Liu, Yuan Jiang, Qiang Li, S. Katharine Hammond

**Affiliations:** 1 Department of Environmental Health Sciences, University of California Berkeley, Berkeley, California, United States of America; 2 National Tobacco Control Office, Chinese Center for Disease Control and Prevention, Beijing, China; 3 Department of Psychology, University of Waterloo, Waterloo, Ontario, Canada; Indiana University School of Medicine, United States of America

## Abstract

**Introduction:**

Smoking is generally not regulated in restaurants or bars in China, or the restrictions are not fully implemented if there are any, while the related hazard health effects are not recognized by the majority of the Chinese population.

**Objectives:**

This study aims to assess the excess health risks and mortality attributed to secondhand smoke (SHS) exposure in restaurants and bars for both servers and patrons to provide necessary evidence for advancing tobacco control in this microenvironment.

**Methods:**

Two approaches were used for the assessment. One is a continuous approach based on existing field measurements and Repace and Lowrey’s dose-response model, and the other is a categorical approach based on exposure or not and epidemiological studies.

**Results:**

Based on the continuous approach, servers were estimated to have a lifetime excess risk (LER) of lung cancer death (LCD) of 730 to 1,831×10^−6^ for working five days a week for 45 years in smoking restaurants and 1,862 to 8,136×10^−6^ in smoking bars, and patrons could have a LER of LCD of 47 to 117×10^−6^ due to visiting smoking restaurants for an average of 13 minutes a day for 60 years, and 119 to 522×10^−6^ due to visiting smoking bars. The categorical approach estimated that SHS exposure in restaurants and bars alone caused 84 LCD and 57 ischemic heart disease (IHD) deaths among nonsmoking servers and 1,2419 LCDs and 1,689 IHD deaths among the nonsmoking patron population.

**Conclusions:**

SHS exposure in restaurants and bars alone can impose high lifetime excess risks of lung cancer death and ischemic heart disease deaths to both servers and patrons, and can cause a significant number of deaths each year in China. These health risks and deaths can be prevented by banning smoking in restaurants and bars and effectively implementing these smoking bans.

## Introduction

More than 600,000 deaths were caused by secondhand smoke exposure (SHSe) in 2004, corresponding to 1.0% of all deaths worldwide in that year [1]. China is the largest consumer of tobacco in the world, with 301 million adults currently smoking (including 53% of men and 2.4% of women) [2] and 556 million (72%) nonsmoking adults exposed to SHS in 2010 [3]. Gan et al. estimated that 130,000 lung cancer deaths (LCDs) and 169,600 ischemic heart disease (IHD) deaths were attributed to active smoking, and 22,200 LCDs and 33,800 IHD deaths were attributed to SHSe among nonsmoking adults in China in 2002 [4].

In China, where the tobacco industry is owned by the government [5], developing and implementing effective policies to reduce active and passive smoking is particularly challenging. China ratified the World Health Organization’s Framework Convention on Tobacco Control (WHO FCTC) in 2006. Health departments of the Chinese government and many health organizations have been working hard to implement the Convention and have made considerable progress. For example, the Beijing government restricted smoking in large restaurants within the city on May 1^st^ 2008; several other big cities, such as Shanghai, Guangzhou, Hangzhou and Yinchuan, have also revised their local legislations to prohibit or restrict smoking in more public places; and the Chinese Ministry of Health revised and tightened the rules of restricting smoking in all indoor public places including restaurants and bars in May 2011; national legislation has been updated to regulate cigarette packaging and labeling and to adjust the consumption tax of tobacco products [5]. However, a huge gap still exists between China’s current state of tobacco control and the FCTC requirements [6], [7]. As reported by Liu, *et al*, Beijing Government’s smoking restrictions are poorly implemented and are ineffective in protecting people from the adverse health effects of SHSe [8].

Another major challenge for tobacco control in China is that SHSe is prevalent in various microenvironments, while the related hazardous health effects are not recognized by nearly 75% of the population [5]. Thus, information about the magnitude of the health risks and disease burden due to SHSe is particularly important for policy makers to plan preventive strategies and for the general population to understand its right of enjoying smoke-free air.

In China, there were about 11 million restaurant or bar employees in 2004 [9], and 15% of people aged 15 years and older eat out at least every day [10]. However, smoking is generally not regulated in hospitality venues, or the restrictions are not fully implemented if there are any [8], [11]. To provide evidence necessary for advancing tobacco control in restaurants and bars, this study aims to assess the health risks and mortality of lung cancer and IHD attributed to SHSe in this specific microenvironment for both servers and patrons.

## Methods

Both a continuous approach based on SHS concentrations and a categorical approach based on exposure or not and epidemiological studies were used for the assessment. Similar approaches have been applied by some other studies [12]–[17].

### Estimate of Life-time Risk of Lung Cancer Death Using a Continuous Approach

Sufficient evidence strongly indicates that SHSe can increase the risk of lung cancer among nonsmokers, and the magnitude of the excess risk is similar among populations with different races/ethnicity, regardless of exposure locations [18]–[20]. Repace and Lowrey [21] developed a model to predict the risk of lung cancer death (LCD) due to SHSe, based on the mortality data and SHSe data among the U.S. population. The model was validated by predicting epidemiologically derived observational data to within 5%, and has been widely used [14]–[16]. The model estimated that, for the U.S. population, the risk of LCD is 5×10^−5^ for exposure to 1 mg/day of SHS particulate matter (SHS PM) for one year and the lifetime excess risk (LER) of LCD can be estimated by Equation 1:
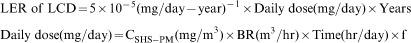
(1)Where

*Daily dose* is the exposure dose of SHS PM in mg/day for an individual;*Y* *ears* is the number of years exposed to SHS;*C_SHS PM_* is the average concentration of SHS PM in mg/m^3^ during the period of exposure;*BR* is the breathing rate in m^3^/hr;*T* *ime* is the average hours per day exposed to SHS;*f* is the adjustment factor, estimated by the number of days per week exposed to SHS divided by 7 days.

Since the long term survival rate of lung cancer is very low [22], the risk of lung cancer death was assumed to be similar to the risk of lung cancer incidence. Since the excess risk of lung cancer caused by SHSe is similar for different populations, the dose-response relationship indicated by Equation 1 was applied to the Chinese population. Servers’ and patrons’ exposures to SHS PM in Chinese restaurants and bars were estimated by existing field measurements of SHS concentrations using particulate matter as the tracer during peak-patronage times in restaurants and bars in some Chinese cities [8], [23]–[27]. Table 1 presents summaries of the results reported by these studies or re-analysis of the data from three of these studies [8], [24], [27]. Three of these studies used a similar protocol and all adjusted the corresponding outdoor PM_2.5_ concentrations to estimate SHS PM concentrations [8], [24], [27]. The other three studies reported indoor PM_2.5_ concentrations only without adjusting the corresponding outdoor levels, and thus were not included in this analysis. Smoking restrictions in restaurants and bars were rare in China before 2008 [11], and the sample sizes of venues restricting or prohibiting smoking in the two existing studies conducted before 2008 [24], [27] were too small to be informative of SHS concentrations in these venues, thus servers’ and patrons’ LER of LCD was estimated based on measurements in venues allowing smoking only. The range of the means of SHS PM concentrations reported by these two studies was used to estimate servers’ and patrons’ exposure to SHS in restaurants and bars before 2008. Measurements conducted in Beijing restaurants and bars in 2008 and 2010 after the implementation of the governmental smoking restriction were used to estimate servers’ and patrons’ potential exposure under poor enforcement.

**Table 1:** Measurements of SHS concentrations indicated by PM_2.5_ in restaurants and bars in some Chinese cities, µg/m^3^.

venue type	city	year	smoking venues			nonsmoking venues			smoking sections			nonsmoking sections		
			n	mean (SD)	median	n	mean (SD)	median	n	mean (SD)	median	n	mean (SD)	median
Restaurants	Beijing	2006 [24]	65	144 (175)	68	11	4 (7)	0				5	18 (17)	22
		2007 [23] a	6	199		6	62					6	131	
		2007 [27]	62	178 (210)	111	7	10 (21)	0				2	4 (5)	4
		2008 [8]	10	57 (62)	33	64	36 (65)	8				13	26 (28)	14
		2008 [25] a,b	20	98	58									
		2010 [8]	18	84 (77)	58	46	70 (159)	27	13	78 (88)	38	17	17 (18)	11
	Guiyang	2007 [27]	63	102 (126)	72	3	28 (49)	0						
	Kunming	2007 [27]	61	71 (104)	45	3	0 (0)	0				1	0	
	Qingdao	2008 [26] a,c										18	29	
												16	99	
	Wuhan	2007 [27]	59	136 (166)	74	2	2 (3)	2				4	17 (23)	8
	Xi’an	2007 [27]	58	157 (179)	90	8	11 (24)	0						
Bars	Beijing	2006 [24]	10	223 (252)	109									
		2007 [23]	6	329										
		2007 [27]	14	195 (269)	44									
		2008 [8]	1	1	1	6	50 (44)	34						
		2010 [8]	13	70 (78)	40	2	280 (395)	280	2	166 (87)	166	3	49 (43)	59
	Guiyang	2007 [27]	14	181 (172)	140									
	Kunming	2007 [27]	13	348 (288)	377							1	945	
	Wuhan	2007 [27]	15	791 (669)	782									
	Xi’an	2007 [27]	14	470 (390)	320							1	12	

Note: SD standard deviation.

^a^ indoor PM_2.5_ concentrations were reported, not adjusted by outdoor PM_2.5_ concentrations;

^b^ 18 restaurants with interventions on training venue staff how to implement smoke-free policies, and the other 16 venues without any interventions.

^c^ 5 restaurants and 15 entertainment venues.

To estimate the daily dose of SHS PM exposure, servers were assumed to have a moderate activity level with a breathing rate of 1.6 m^3^/hour, and patrons were assumed to have a light activity level with a breathing rate of 1.0 m^3^/hour, based on recommendations by U.S. EPA [28]. Servers’ exposure time was assumed to be four hours a day during peak patronage time for five days a week, and a working life of 45 years was also assumed. For patrons, an average adult was reported to spend 13 minutes per day (male 16 minutes, female 9 minutes) in restaurants and bars in 2008 [31]. Based on these assumptions, *f* equals to 5days/7days for servers and one for patrons.

### Estimate of Health Risk by Excess Risk Assessment Method (Exposed/Nonexposed)

This approach uses the population attributable fraction (PAF) to estimate the attributable deaths of lung cancer and ischaemic heart disease due to SHSe in restaurants and bars. PAF is referred as the proportional reduction in disease that would occur if the exposure was reduced to zero. This approach has been commonly used to assess disease burden attributed to SHSe worldwide and in some individual countries [1], [4], [32], [33]. Equations 2 and 3 were used for the assessment.
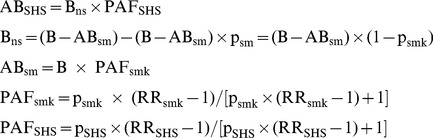
(2)

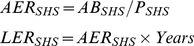
(3)Where

*B* is the total number of deaths of a disease per year among the whole population;*B_ns_* is the number of deaths of a disease per year among nonsmokers;*AB_sm_*_,_ is the attributable number of deaths of a disease per year among smokers due to smoking;*AB_SHS_* is the attributable number of deaths from a disease per year among nonsmokers due to SHSe;*PAF_smk_* is the PAF of the total deaths of a disease among a population due to active smoking;*PAF_SHS_* is the PAF of the deaths of a disease due to SHSe among nonsmokers;*p_smk_* is the prevalence of smoking;*p_SHS_* is the prevalence of SHSe;*RR_smk_* is the relative risk of a disease due to active smoking;*RR_SHS_* is the relative risk of a disease due to SHSe among nonsmokers;*AER_SHS_* is the average annual excess risk of death due to SHSe among nonsmokers;*P_risk_* is the population at risk, that is, nonsmokers aged 35 years or older;*LER_SHS_* is the life time excess risk of death due to SHSe among nonsmokers;*Y* *ears* is the number of years of exposure during lifetime.

China made up of 82% of the population of WHO WPRO-B region (World Health Organization Western Pacific Regional Office countries with low child and adult mortality rates) in 2004, thus this factor was applied to the WPRO-B subregion data to estimate the age and gender specific population and mortality (*B* in the equations above) of lung cancer and ischemic heart disease (Table 2). Because of the latency of developing lung cancer or ischemic heart disease, the calculations were restricted to adults aged above 30 years.

**Table 2:** Current smoking prevalence and SHS exposure prevalence by gender and age in 1984, 1996 and 2002 in China.

	Population^a^		IHD	hospitality	smoking prevalence^d^			SHS exposure prevalence^e^
	Population^a^	LCD^b^	death^c^	employees	1984	1996	2002	1996
**total**	1,299,880,000	348,469	731,647	11,283,747	34%	35%	36%	54%
**male**	667,869,966	240,136	379,430	5,021,199	61%	63%	66%	46%
15–30	328,001,739	659	4,641	1,775,729	37%	53%	49%	50%
30–44	169,321,861	10,760	14,100	2,237,217	68%	74%	67%	46%
45–59	105,910,826	55,405	55,662	943,042	72%	73%	71%	46%
60+	64,635,539	173,971	309,668	65,210	67%	69%	69%	38%
**female**	632,010,034	108,333	352,217	6,262,549	7.0%	4.2%	3.1%	57%
15–30	300,237,820	331	1,653	2,225,430	0.8%	1.0%	1.0%	57%
30–44	161,802,513	6,183	9,004	3,096,796	5.5%	2.7%	2.5%	63%
45–59	101,349,079	23,360	27,456	915,247	16%	6.9%	4.8%	57%
60+	68,620,622	78,790	315,757	25,075	18%	9.4%	7.8%	46%

Note: ^a^the Chinese population was 1,299,880,000, reported by the National Bureau of Statistics; the population by gender and age groups were estimated from the WHO WPR-B dataset with a proportion of 82% (total Chinese population/total WHO WPR-B population in 2004) because they were not available from the bureau’s datasets; The numbers in each of the first four columns may not add up to the corresponding subtotals or totals due to rounding up during calculation.

^b^ LCD, lung cancer deaths, estimated from the WHO WPR-B dataset;

^c^ IHD, ischaemic heart disease death, estimated from the WHO WPR-B dataset;

^d,e^ data from a national survey in 1984 [36], in 1996 [35] and in 2002 [38].

There were 11,285,000 people employed by restaurants and bars in 2004 [34]; and gender and age distributions of restaurant and bar employees in urban areas (but not in rural areas) in 2004 could be obtained from the China Labor Statistical Yearbook [29] (Table 2). For this analysis, the gender and age distributions of all restaurant and bar employees were assumed to be the same as those of urban employees. The mortality rates of lung cancer and ischemic heart disease for this specific population was not available, thus, they were assumed the same as among the general population.

Age and gender specific smoking prevalence was obtained from the 1984, 1996 and 2002 national surveys of Chinese adult smoking behaviors [35]–[38] (Table 2). Detailed information on SHSe was available from the 1996 survey only; since the smoking prevalence of Chinese male adults did not change much from 1984 to 2002, SHSe prevalence in 1996 was used in this analysis. To account for the latency of lung cancer, the 1984 smoking prevalence and the 1996 SHSe prevalence of the 2004 population were used to calculate the PAF. For IHD death estimates, the average of the smoking prevalence in 1984, 1996 and 2002 was used to account for both the short-term and long-term effect of smoking on the disease. About 90% of restaurants and bars patrons reported that they were exposed to SHS in those places in 2010 [3], and this percentage was expected to be higher in earlier times, e.g. before 2004. For this analysis, 95% of restaurant and bar patrons as well as workers were assumed to be exposed to SHS in this microenvironment.

The relative risks (*RRs*) of lung cancer and ischaemic heart disease used to calculate the PAF due to active smoking and SHSe were the same as used by Gan et al [4]. The relative risks of these two diseases for different ages were unavailable, and they were assumed the same across all age groups.

Homes, workplaces, and indoor public places, including restaurants and bars, were assumed to be the major microenvironments where people were exposed to SHS. To estimate the deaths of the two diseases due to SHSe in restaurants and bars, the number of total deaths attributed to SHSe in all microenvironments was multiplied by the percentage of SHS time in this microenvironment, which was estimated by weighting the proportion of the population of interest with SHSe in different microenvironments by their average time spent in each microenvironment. The proportion of males or females exposed to SHS in a specific microenvironment was estimated by multiplying the corresponding general prevalence of SHSe by the proportion of passive smokers who had SHSe in that specific environment as reported in 1996 [35]. The average time spent in each microenvironment was estimated based on time activity surveys conducted in 10 provinces across China in 2008 [31]. Restaurant and bar workers were assumed to spend similar time in homes, workplaces, and indoor public places as the general population, while their time spent in restaurants and bars as patrons was omitted. People were assumed to be exposed to SHS in residences only during waking hours. The percentage of SHS time for each type of microenvironment was calculated as.
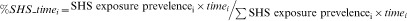
(4)Where

*i* is the *i*th microenvironment, which includes home, workplace, restaurants and bars, and other indoor public places;*%SHS_time_i_* is the fraction of time exposed to SHS in the *i*th microenvironment of the overall time exposed to SHS in all microenvironments by the population;*SHS exposure prevalence_i_* is the proportion of the nonsmoking population exposed to SHS in the *i*th microenvironment;*T* *ime_i_* is the time spent in the *i*th microenvironment.

Sensitivity analysis of percentage of SHSe time in restaurants and bars was conducted by varying the smoking policy in different microenvironments. With this approach, the significance of SHSe in restaurants and bars was examined, in terms of the fraction of overall SHSe time for restaurant and bar servers and patrons.

## Results

### Estimate of Life-time Risk of Lung Cancer Death Using a Continuous Approach

Based on field measurements in 368 smoking restaurants and 80 smoking bars in five Chinese cities in 2006 and 2007 [24], [27], servers were estimated to have an lifetime excess risk (LER) of lung cancer death (LCD) of 730 to1,831×10^−6^ for working five days a week for 45 years in smoking restaurants and 1,862 to 8,136×10^−6^ in smoking bars; and patrons were estimated to have LER of LCD of 47 to 117×10^−6^ for visiting smoking restaurants for an average of 13 minutes a day for 60 years, and 119 to 522×10^−6^ due to visiting smoking bars (Table 3). After the implementation of the governmental smoking restrictions in Beijing restaurants and bars in 2008, the mean SHS PM concentrations during peak-patronage time ranged from 57 to 84 µg/m^3^ in restaurants (n = 28) that allow smoking, approximately 78 µg/m^3^ in designated smoking sections (n = 13), 17 to 26 µg/m^3^ in designated nonsmoking sections (n = 30), and 36 to70 µg/m^3^ in nonsmoking restaurants (n = 110) [39] (Table 1). If future smoking restrictions are as poorly enforced as the 2008 Beijing governmental smoking restrictions, and servers and patrons are exposed to similar SHS concentrations as measured in Beijing restaurants in 2008 and in 2010 for a lifetime, servers’ lifetime excess risk of LCD could range from 370 to 720×10^−6^ due to work in nominal nonsmoking restaurants, and 175 to 802×10^−6^ due to work in restaurants nominally restrict smoking, and patrons could have lifetime excess risk ranged from 24 to 46×10^−6^ due to visit nominal nonsmoking venues only or from 11 to 17×10^−6^ due to visiting designated nonsmoking sections only for a lifetime.

**Table 3:** Estimates of lifetime excess risk of lung cancer deaths based on the continuous approach in Chinese restaurants and bars.

	smoking restaurants		smoking bars	
	servers	patrons	servers	patrons
Range of mean concentrations, µg/m^3a^	71–178	71–178	181–791	181–791
Dose response	5×10^−5^ per year for exposure to 1 mg SHS PM per day			
Breathing rate, m^3^/hr^b^	1.6	1	1.6	1.0
Average time exposed to SHS, hr/day^c^	4	0.22	4	0.22
Number of days exposed to SHS, day/week^c^	5	7	5	7
Number of years exposed to SHS, years	45	60	45	60
Lifetime excess risk of lung cancer death, 10^−6^ d	730–1,831	47–117	1,862–8,136	119–522

Note: ^a^Range of mean concentrations were estimated from field measurements in restaurants and bars in five Chinese cities in 2007 and in Beijing 2006;

^b^ breathing rates of different levels of activities specific for Chinese populations were not available and they were assumed according to recommendations by U.S. EPA [28];

^c^ servers’ exposure to SHS at work were assumed to occur mostly in four hours during peak patronage time on each of the five working days; patrons’ exposure time was based on the report that people spend an average of 13 minutes (0.22 hours) a day in restaurants and bars every day [31];

^d^ the lifetime attributable risk was calculated using the dose-response relationship estimated by Repace and Lowrey [46] (Eq.1).

### Estimate of Health Risk by Excess Risk Assessment Method (Exposed/Nonexposed)

The time servers spend exposed to SHS in their work places (restaurants and bars) was estimated to account for 85% of their total SHS exposure time for males and 62% for females (Table 4), which corresponded to 28 LCDs and 19 IHD deaths for males and 56 LCDs and 38 IHD deaths for females in 2004 (Table 5). Patrons’ exposure to SHS due to visiting smoking restaurants and bars accounted for 9.6% of their total exposure time for males and 3.2% for females (Table 4), corresponding to 743 LCDs and 673 IHD deaths among males and 676 LCDs and 1,016 IHD deaths for females in 2004 (Table 5). A total of 3,249 deaths from lung cancer and IHD were attributed to SHSe in restaurants and bars for the servers and patrons together. The LER for nonsmokers were estimated to be 762×10^−6^ for LCD and 524×10^−6^ for IHD deaths among servers who work in restaurants and bars for 45 years and 200×10^−6^ for LCD and 240×10^−6^ for IHD among patrons who visit restaurants and bars for 60 years.

**Table 4:** Estimated percentage of total SHS exposure happening in different types of environments for restaurant and bar servers and patrons by gender.

	Male			Female		
	SHS exposure prevalence	time spent (minutes)	%SHS-time	SHS exposure prevalence	time spent (minutes)	%SHS-time
servers						
home^a^	19.6%	380	14.4%	46.7%	493	37.0%
work place^b^	95.0%	461	85.0%	95.0%	409	62.4%
indoor public places^c^	18.2%	17	0.6%	16.0%	23	0.6%
patrons						
home^a^	19.6%	380	46.8%	46.7%	493	86.0%
work place	20.5%	324	41.7%	10.8%	234	9.5%
indoor public places^c^	18.2%	17	1.9%	16.0%	23	1.4%
restaurants and bars	95.0%	16	9.6%	95.0%	9	3.2%

Note: SHS exposure prevalence is the proportion of nonsmokers exposed to SHS;

^a^ people were assumed to be exposed to SHS in residences only during waking hours;

^b^ for servers, the work place was restaurants and bars, and 95% of Chinese restaurants and bars were assumed to allow smoking;

^c^ indoor public places did not include restaurants and bars in this analysis.

**Table 5:** Estimated numbers of lung cancer death and ischaemic heart disease death due to SHS exposure in restaurants and bars based on excess risk assessment methods.

	total deaths among the population	deaths attributable	deaths among nonsmokers	deaths attributable to overall SHS	deaths attributable to SHS exposure inrestaurants and bars		
	age >30	to smoking	aged >30	exposure	workers	patrons	total
male							
LCD	240,136	130,085	34,419	7,800	28	743	771
IHD	379,430	127,634	76,301	7,060	19	673	692
female							
LCD	108,333	18,148	79,119	21,237	56	676	732
IHD	352,217	43,997	263,775	31,872	38	1016	1054
Total							
LCD	348,469	148,234	113,538	29,038	84	1,419	1,503
IHD	731,647	171,630	340,076	38,932	57	1,689	1,746

Note: LCD: lung cancer death; IHD: ischaemic heart disease.

When 95% of restaurants and bars allow smoking, working in restaurants and bars accounts for almost all of their exposure time for servers living in smoke-free homes, and for more than half of their total exposure time for servers living in smoking homes. For patrons who are exposed to SHS at home, their exposure time in restaurants and bars accounted for less than 5% of their total exposure time, regardless of the smoking policy in their workplaces. However, for patrons who do not have any SHSe at homes or workplaces, their exposure time in restaurants and bars dominate their total exposure time even if only 50% of restaurants and bars allow smoking (Table 6).

**Table 6:** Sensitivity analysis of percentage of overall SHS time occurred in restaurants and bars for workers and patrons.

SHS exposure prevalence			servers %SHS-time		patrons %SHS-time	
home	work places (patrons)	restaurants and bars	male	female	male	female
Yes	yes	95%	53%	44%	3.3%	1.6%
yes	yes	80%	49%	40%	2.8%	1.4%
yes	yes	50%	38%	29%	1.7%	0.9%
yes	yes	20%	19%	14%	0.7%	0.3%
yes	no	95%	53%	44%	3.8%	1.7%
yes	no	80%	49%	40%	3.2%	1.4%
yes	no	50%	38%	29%	2.0%	0.9%
yes	no	20%	19%	14%	0.8%	0.4%
no	yes	95%	99%	99%	18%	23%
no	yes	80%	99%	99%	16%	20%
no	yes	50%	99%	98%	10%	13%
no	yes	20%	97%	96%	4.0%	6.0%
no	no	95%	99%	99%	83%	70%
no	no	80%	99%	99%	81%	66%
no	no	50%	99%	98%	72%	55%
no	no	20%	97%	96%	51%	33%

## Discussion

Due to limited information available to assess the health risks of other diseases due to SHSe in China, including acute respiratory and sensory irritation, adult asthma initiation, breast cancer among young females, etc., only lung cancer deaths (LCDs) and ischemic heart disease (IHD) deaths were included in this analysis. Therefore, the total health risk and mortality due to SHSe in Chinese restaurants and bars is expected to exceed the estimates in this study.

Several studies have used the dose-response relationship reported by Repace and Lowrey [21] to estimate lifetime excess risk (LER) of LCD due to SHSe by hospitality workers. Lopez et al. [15] estimated a LER of LCD of 1,450×10^−6^ for hospitality workers working for 40 years in Spain; Siegel and Skeer [16] estimated a LER of 4,100×10^−6^ for U.S. bar workers for a working life of 45 years, and Liu et al. [12] estimated a LER of 802 (658–936)×10^−6^ for Minnesota restaurant and bar workers. This analysis estimated the LER of LCD for servers of Chinese restaurants and bars for the first time. The estimates for restaurants servers (a LER of 730–1,831×10^−6^ for servers working for 45 years in smoking restaurants and 1,862–8,136×10^−6^ in smoking bars) were similar to the estimates for Spain and U.S. hospitality workers [15], [16] and higher than that for Minnesota restaurants and bars. All these estimated LCD risks for servers are much higher than the *de manifestis risk* of 3×10^−4^, a risk of obvious or evident concern, and one that U.S. public agencies will almost always regulate to control, or mitigate when recognized [40]. Most estimated LCD risks are also higher than the significant risk of 1×10^−3^ defined by the U.S. Occupational Safety & Health Administration (OSHA).

Few studies in literature have assessed health risks for patrons. Liu et al. [12] and this study are the first to do so. Liu et al. [12] estimated a LER of LCD of 80 (66–95)×10^−6^ for Minnesota patrons visiting smoking restaurants and bars or sections only and 18 (13–23)×10^−6^ for those visiting designated nonsmoking sections of restaurants and bars only for 1.4 hours/week for 60 years; this analysis estimated a LER of 47 to117×10^−6^ due to visiting smoking restaurants for an average of 13 minutes a day (1.5 hours/week) for 60 years, and 119 to 522×10^−6^ due to visiting smoking bars. Analyses of the attendant LCD risk due to SHSe in Minnesota restaurants and bars were based on measurements of SHS concentrations in a representative sample, with restaurants and bars combined and exposure from smoking venues and designated smoking sections combined, while analyses in this study were based on SHS measurements in convenience samples of smoking venues. These estimated lung cancer death risks are all much higher than the acceptable risk or *de minimis risk* of 1×10^−6^ for large populations [40].

Based on the attributable risk assessment method, about 29,000 LCDs and 38,900 IHD deaths were attributable to SHS in 2004; about 30% and 15%, respectively, higher than estimates by Gan et al. [4], which were 22,200 LCDs and 33,800 IHD deaths. The reasons may be that the 2004 population of WHO WPRO-B was 1% higher than the 2002 population, and the total LCDs and IHD deaths in 2004 were 18% and 4%, respectively, higher than in 2002; furthermore, the population was divided into more subgroups in the analysis by Gan et al. than in this analysis, which may also lead to some discrepancy between the two analyses.

Jamrozik [33] estimated a total of 54 deaths of lung cancer, IHD and stroke among restaurant and bar workers due to their SHSe at work in the United Kingdom; Liu et al. [12] estimated a total of 3,200 deaths of lung cancer and ischaemic heart disease among hospitality workers and patrons due to SHSe in restaurants and bars in the U.S., based on the 1992–1994 U.S. *National Human Activity Pattern Survey* [41] that SHSe accounted about 9% of total SHSe in the U.S.; this analysis estimated a total of 3,249 deaths of the two diseases among hospitality servers and patrons caused by SHSe in restaurants and bars, accounting for about 5% of the total LCDs and IHD deaths attributed to SHS for the whole nonsmoking population. The total number of excess deaths of LCD and IHD caused by SHSe in restaurants and bars alone in China (3,249) is comparable to the total deaths from all sexually transmitted diseases, excluding HIV, among those aged ≥30 years, or to the total deaths from skin diseases (3,310 and 3,586, respectively, in the entire WHO WPRO-B region, with 82% estimated to be in China). While reducing the disease burden of the later two types of diseases is very expensive and challenging, eliminating SHSe in restaurants and bars, and thus preventing a similar number of deaths, is much more cost effective and practical.

About 20% of nonsmoking males are exposed to SHS at home and 21% in workplaces; about 47% of nonsmoking females are exposed to SHS at homes and 11% in workplaces in China. This leaves 63% of nonsmoking males and 47% of nonsmoking females who are not exposed to SHS in either their homes or workplaces. For this population, their time exposed to SHS in restaurants and bars accounts for more than 66% of their total exposure time, if 80% of the restaurants and bars allow smoking, which is true in most Chinese cities before 2011. Restaurants and bars are the most significant source of SHSe for more than half of the nonsmoking adult population. If smoking regulations are not fully implemented as happened with the 2008 Beijing governmental smoking restrictions (after which implementation, smoking was still observed in about half of nonsmoking venues or sections [39]), the time spent in restaurants and bars still dominates the total SHS exposure time for patrons who live in smoke-free homes and work in smoke-free environments, and for nonsmoking servers who live in smoke-free homes. Thus, passing smoke-free legislation is not enough, and effective implementation is necessary to protect against SHSe.

The lifetime excess risks and the number of LCD and IHD deaths caused by SHSe in restaurants and bars are probably underestimated for several reasons. Zheng et al. [30] reported that restaurant workers in Shanghai, China were exposed to SHS during working hours for 24.2 hours on average per week, while this analysis only assumed 20 hour/week of SHSe for workers when using the continuous dose-response approach. When using the attributable risk approach, exposure intensity (indicated by SHS concentrations) in different microenvironments was not weighted, while SHS concentrations are usually higher in restaurants and bars than in homes and most other work places: in the U.S. before 1999, mean airborne nicotine concentrations ranged from 2 to 6 µg/m^3^ in smoking offices, from 1 to 6 µg/m^3^ in smoking workplaces of blue-collar workers, from 1 to 3 µg/m^3^ in homes of smokers, and from 3 to 8 µg/m^3^ in restaurants [42], which have been considerably higher concentrations. In public places in some rural and urban areas in China, the medians of one-week time-weighted average concentrations were reported to range from 0.1 to 0.7 µg/m^3^ in schools, hospitals, governmental buildings, and transportations, and was 2.2 µg/m^3^ in restaurants [43]; a median of week-long nicotine concentrations of 1.2 µg/m^3^ in 14 office buildings in China was also reported [44]; the median of week-long nicotine concentrations in some Chinese homes was found to be about 0.2 µg/m^3^ [45]. If these median nicotine concentrations in different environments in China reported in previous studies were used to weight the exposure time, the number of LCD and IHD deaths caused by SHSe in restaurants and bars would be 40% higher for servers and three times higher for patrons.

It should be noted that results presented in this paper should be interpreted with caution. Although the models used in this paper have been used by many other researchers, no validation exists for the Chinese worker and patron population in the hospitality industry; and the Repace-Lowrey dose-response relationship and the inhalation rates used in the analysis are based on U.S. data. However, the authors believe that the most relevant approaches and data are used in this analysis, and no efforts have been made to exaggerate the toll of diseases due to SHSe in Chinese restaurants and bars.

## Conclusions

SHS exposure in restaurants and bars alone can impose high lifetime excess risks of lung cancer death and ischaemic heart disease deaths to both servers and patrons, and can cause a significant number of deaths each year in China. These health risks and deaths can be prevented by banning smoking in restaurants and bars and effectively implementing these smoking bans.
